# Pulmonary edema during cesarean delivery secondary to Takotsubo syndrome revealing pheochromocytoma: a case report

**DOI:** 10.1016/j.xagr.2026.100700

**Published:** 2026-09-26

**Authors:** Adnane Berdai, Djoudline Doughmi, Said Benlamkaddem, Mustapha Harandou

**Affiliations:** Mother-Child Anesthesia and ICU Department, Faculty of Medicine, Pharmacy, and Dental Medicine, Hassan II University Hospital, Sidi Mohamed Ben Abdellah University, 30 000, Fez, Morocco

**Keywords:** cesarean section, noninvasive ventilation, pheochromocytoma, point-of-care ultrasound, pulmonary edema, takotsubo syndrome

## Abstract

Acute pulmonary edema during delivery is a serious condition with a wide range of etiologies, including Takotsubo syndrome (TTS), a form of transient left ventricular dysfunction most often presenting as apical ballooning. We report the case of a 35-year-old woman who developed severe hypertension (205/130 mmHg), polypnea, and a drop in oxygen saturation to 80% during cesarean delivery under spinal anesthesia. She received noninvasive ventilation while the cesarean section continued and was transferred to the intensive care unit. Point-of-care ultrasound showed a left ventricular ejection fraction (LVEF) of 35% with apical ballooning and diffuse B-lines on lung ultrasound. Computed tomography identified a right adrenal mass. A diagnosis of pulmonary edema complicating TTS was made, and the patient received furosemide and oxygen therapy. Further investigations, including urinary catecholamine metabolites, confirmed the diagnosis of pheochromocytoma. One week later, LVEF had recovered to 57% with mild residual apical hypokinesia and complete clinical recovery. The patient was then transferred to the surgical unit for management of the pheochromocytoma. This case demonstrates that TTS can cause pulmonary edema in the peripartum period, and that timely diagnosis is essential for a favorable outcome. Point-of-care ultrasound can be instrumental in guiding the assessment of such critical situations, and noninvasive ventilation may be of particular value in selected pregnant patients. This case also underscores the importance of an etiological workup and prompt treatment of hypertensive disorders in pregnancy, especially when pheochromocytoma is suspected, since anesthesia and surgery can precipitate decompensation.

## Introduction

Acute pulmonary edema is a significant cause of morbidity and mortality during pregnancy. Its estimated incidence ranges from 0.08% to 0.5%.^1^ It represents the final clinical manifestation of a wide range of underlying aetiologies and pathophysiological mechanisms, and its rapid recognition is critical given the need for timely intervention in a pregnant or peripartum patient.

Takotsubo syndrome (TTS), or stress cardiomyopathy, is an acute and typically reversible form of left ventricular dysfunction triggered by intense emotional or physical stress and catecholamine excess.^2^ Although increasingly recognized in the general population, TTS remains exceptionally rare in the peripartum period, where its presentation can be difficult to distinguish from other causes of acute cardiac decompensation such as peripartum cardiomyopathy or pre-eclampsia-related pulmonary edema. Pheochromocytoma, an equally rare catecholamine-secreting tumor, can independently precipitate TTS through massive catecholamine surges, particularly around the time of anesthesia or surgery, and this dual rarity makes such presentations especially prone to diagnostic delay.

We report a case of acute pulmonary edema during Caesarean delivery secondary to TTS triggered by an undiagnosed pheochromocytoma. Outcome can be improved in similar patients by early identification and an appropriate diagnosis of the underlying etiology.

## Case description

A 35-year-old apparently healthy woman, gravida 4 para 3, with an uneventful pregnancy and no significant medical history or known allergies, was scheduled for cesarean section because of three previous cesarean deliveries. She had fasted for 6 hours before surgery. On admission, her heart rate was 87 beats/min, SpO₂ 98%, and blood pressure 132/83 mmHg. Following crystalloid co-loading, spinal anesthesia was performed with 12.5 mg of hyperbaric bupivacaine. Progressively after the initiation of spinal anesthesia, the patient complained of chest pain and discomfort. The upper sensory block level was T5, and no clinical signs of a high or total spinal block were observed. She subsequently developed polypnea, tachycardia, and became hypoxemic. The pulsed saturation of oxygen decreased to 80%. Meanwhile, the parturient developed severe hypertension (205/130 mmHg), remained conscious, and had bilateral crackles on auscultation. No prophylactic or rescue vasopressors had been administered. As the cesarean delivery continued, she received noninvasive ventilation (NIV) through bilevel positive airway pressure. She remained conscious and cooperative throughout, with preserved airway-protective reflexes, allowing NIV to be safely continued under close monitoring. The oxygen saturation progressively improved to 100%. The neonate had an Apgar score of 10 at both 1 and 5 minutes, and physical examination was unremarkable.

At the end of the cesarean section, clinical evaluation confirmed persistent polypnea. The patient was transferred to the intensive care unit for additional support. At admission, lung ultrasound showed diffuse bilateral B-lines, while transthoracic echocardiography (TTE) showed a left ventricular ejection fraction (LVEF) of 35% with apical ballooning. There was no right ventricular dilatation and no pericardial effusion.

Thoracic computed tomography scan ruled out pulmonary embolism and confirmed pulmonary edema. Troponin I level was initially 0.89 ng/mL and NT-proBNP was 2992 pg/mL. The electrocardiogram showed sinus tachycardia. There was no evidence of tissue hypoperfusion, with a lactate level of 1.87 mmol/L. Arterial blood gas showed respiratory alkalosis: pH 7.55, PaCO2 29 mmHg, HCO3- 23 mmol/L.

Pre-eclampsia was initially considered because of the severe hypertension and pulmonary edema. However, the pregnancy had been uneventful, with no antenatal history of hypertension or proteinuria. At the time of the acute event, proteinuria was absent, and platelet count and liver enzymes were normal, making this diagnosis less likely.

Based on the clinical, laboratory, echocardiographic, and chest computed tomography findings, a diagnosis of pulmonary edema secondary to Takotsubo syndrome (TTS) was made. The patient was treated with intravenous furosemide 20 mg and oxygen administered via a face mask at 6 L/min.

During her intensive care stay, the patient received dobutamine at a maximum of 5 µg/kg/min, oxygen by mask, and enoxaparin at prophylactic dosing. On day 3, she was successfully weaned from oxygen therapy and dobutamine, with disappearance of polypnea and normalization of troponin levels. She had normal tissue perfusion and urine output.

Further investigations, including abdominal ultrasound and magnetic resonance imaging, demonstrated a 12 × 10 cm right adrenal mass (Figure). A 24-hour urine total normetanephrine, metanephrine, and 3-orthomethyldopamine measurement was respectively 9450 mmol/day (normal range [NR] < 281 lt; 281 mmol/day), 1485 mmol/day (NR < 159), and 1090 mmol/day (NR < 329). Hemogram, serum electrolytes, cortisol, and thyroid hormone dosing were within normal range. This confirmed the diagnosis of pheochromocytoma that triggered TTS in the setting of cesarean delivery, neuraxial anesthesia, and perioperative drug administration.

**Figure:**
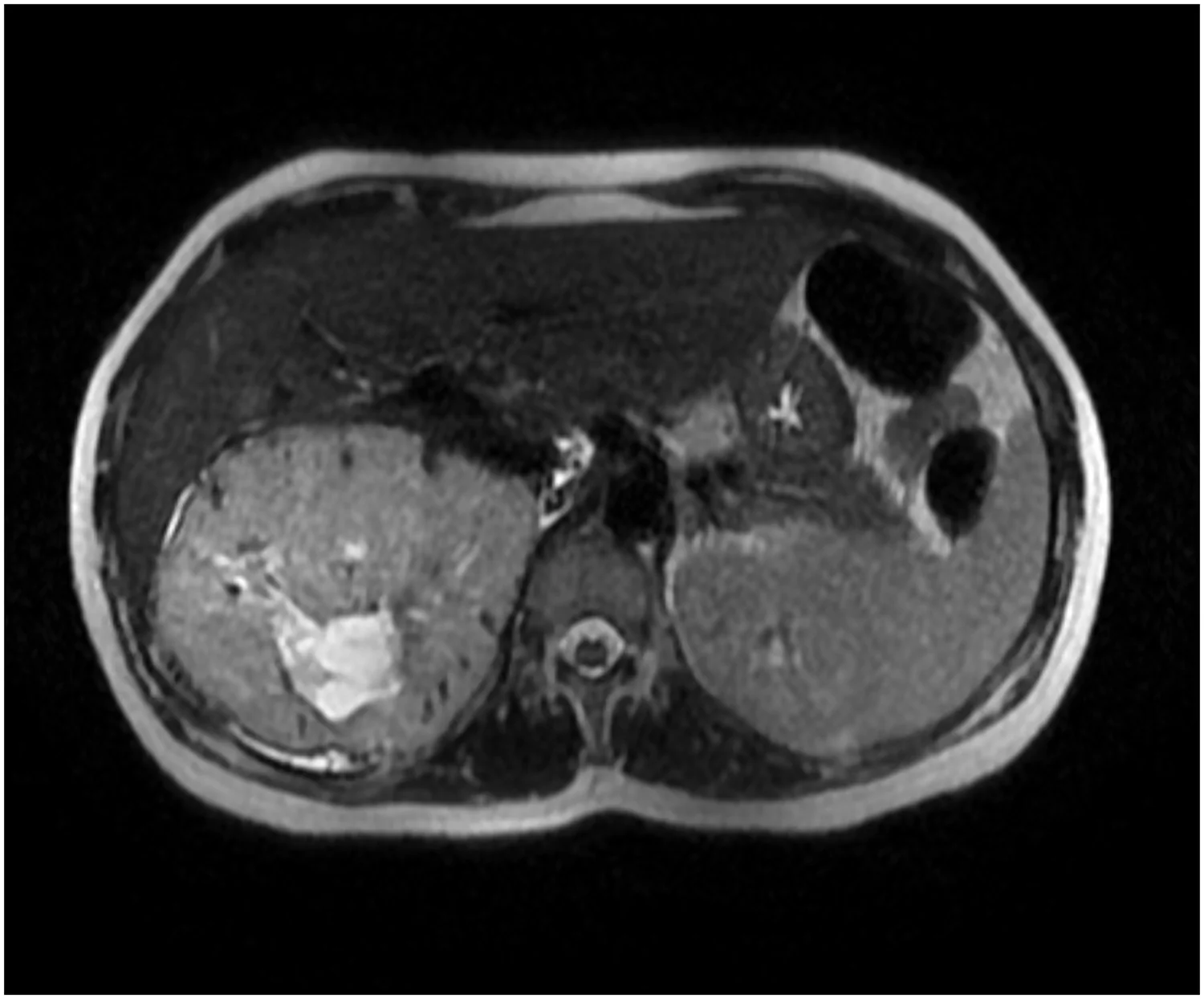
Abdominal magnetic resonance imaging demonstrating a large heterogeneous right adrenal mass

Berdai. Pulmonary edema during cesarean delivery secondary to Takotsubo syndrome revealing pheochromocytoma. Am J Obstet Gynecol 2026.

Serial TTE demonstrated progressive recovery of left ventricular function. On day 7, LVEF was 57% with persistence of mild apical hypokinesia. The patient was then transferred to the surgery unit for management of the adrenal mass. A TTE performed 6 months after delivery showed complete recovery of left ventricular function.

## Discussion

Acute respiratory failure (ARF) is one of the leading causes of intensive care unit admission in pregnant women.^3^ The most common underlying etiologies include cardiogenic and noncardiogenic pulmonary edema, pneumonia, and pulmonary embolism.^4^ In the obstetric setting, point-of-care ultrasound (POCUS) is an immediately available, noninvasive tool that can rapidly identify the cause of acute dyspnoea or hypotension, guide resuscitation, and assess cardiac function, particularly when specialist consultation is not readily accessible.^5^^,^^6^ Moreover, multiorgan POCUS has a substantial diagnostic and therapeutic impact in critically ill pregnant patients, helping to differentiate between cardiac, pulmonary, or mixed etiologies of dyspnea or ARF.^7^ In our case, diffuse B-lines on lung ultrasound, together with apical hypokinesia on echocardiography, were consistent with a cardiogenic origin of the pulmonary edema, which we believe resulted from the combination of TTS and severe hypertension secondary to pheochromocytoma.

TTS, also called stress cardiomyopathy, is characterized by transient left ventricular dysfunction with distinct regional wall-motion abnormalities, most commonly apical ballooning, occurring in the absence of obstructive coronary artery disease or infectious myocarditis.^8^ The term “takotsubo” translates from Japanese as “octopus pot,” referring to the characteristic ballooning shape of the left ventricular apex.^9^ Our patient’s clinical and imaging findings, including moderately elevated troponin and significantly elevated NT-proBNP levels in the absence of infectious myocarditis or obstructive coronary artery disease, were consistent with this pattern and fulfilled the International Takotsubo Diagnostic Criteria^2^ and were confirmed by an independent cardiologist.

Postmenopausal women are particularly susceptible, and the condition generally carries a favorable prognosis.^10^ TTS remains extremely rare in the peripartum period, with the first case described in 2008,^11^^,^^12^ and is often difficult to differentiate from peripartum cardiomyopathy (PPCM),^13^ which is driven by prolactin-mediated inflammation in the setting of a genetic predisposition.^14^ TTS and PPCM can share electrocardiographic and biomarker features, and the primary distinguishing feature in most studies remains the characteristic echocardiographic appearance.^15^

Distinguishing TTS from PPCM in our patient relied on both echocardiographic phenotype and risk-factor analysis. High parity is a recognized PPCM risk factor, but she lacked other established risk factors such as multifetal gestation, obesity, or assisted reproduction; her rapid, near-complete recovery of LVEF within one week, together with the apical ballooning, was more consistent with TTS than with the typically more protracted recovery of PPCM.^14^^,^^15^ Peripartum TTS also shows a higher prevalence of atypical presentations, less ST-segment elevation, and lower LVEF than TTS in the general population, while maintaining an excellent mid- and long-term prognosis.^16^

TTS is usually triggered by an emotional or physical stressor associated with catecholamine excess.^2^ Reported triggers include pregnancy-related physiological stress, exogenously administered catecholamines, and catecholamine-secreting tumors such as pheochromocytomas and paragangliomas; the absence of exogenous catecholamine administration in our patient supports the pheochromocytoma as the most likely trigger.^13^^,^^17^ In patients with asymptomatic pheochromocytoma, spontaneous or procedure-related catecholamine surges may precipitate TTS and other severe cardiovascular complications.^1^ This diagnosis was confirmed by MRI and the marked elevation of all catecholamine metabolites—normetanephrine, metanephrine, and 3-orthomethyldopamine.

Management of ARF in this setting is particularly challenging. In our patient, NIV successfully stabilized oxygenation and avoided endotracheal intubation. NIV was historically avoided in pregnancy because of a theoretical aspiration risk, but in carefully selected obstetric patients who remain alert and able to protect their airway, it represents a safe and effective option for acute respiratory failure,^4^ particularly suited to rapidly reversible respiratory disorders while avoiding complications associated with invasive mechanical ventilation.^18^^,^^19^

This decision was supported by several favorable criteria: the patient remained alert and able to protect her airway, tolerated the ventilatory interface, and showed a rapid improvement in oxygenation within minutes of NIV initiation, all of which are recognized prerequisites for safe NIV use in the obstetric population.^20^ Safety was further ensured by a pre-established strategy anticipating escalation to endotracheal intubation in the event of altered consciousness, major NIV intolerance, or persistent SpO2 <90% despite NIV.

Finally, the management of TTS during pregnancy requires a multidisciplinary approach, with supportive therapy remaining the cornerstone of treatment.^15^ Although recovery of cardiac function typically occurs within 4 weeks, some patients require long-term heart failure management.^13^ In such cases, a subsequent pregnancy should be followed appropriately but not systematically contraindicated. In the peripartum period, triggers that can favor or induce this cardiomyopathy should be avoided. Epidural analgesia and anesthesia can play an important role in such cases.

## CRediT authorship contribution statement

**Adnane Berdai:** Writing – review & editing, Writing – original draft, Supervision, Project administration, Conceptualization. **Djoudline Doughmi:** Writing – review & editing, Writing – original draft. **Said Benlamkaddem:** Writing – review & editing, Conceptualization. **Mustapha Harandou:** Writing – review & editing, Conceptualization.
